# A comparison of different irrigation systems and gravitational effect on final extrusion of the irrigant 

**DOI:** 10.4317/jced.52158

**Published:** 2015-04-01

**Authors:** Emel Uzunoglu, Melahat Görduysus, Ömer Görduysus

**Affiliations:** 1 Department of Endodontics, Faculty of Dentistry, Hacettepe University, Ankara, Turkey

## Abstract

**Background:**

The aim of this study was to compare manual needle irrigation (MNI), RinsEndo (RE), and passive ultrasonic irrigation (PUI), and assess the effect of gravity on extrusion from the apex in vitro.

**Material and Methods:**

The distobuccal roots of molars were used and the canals were instrumented up to F2. Teeth were mounted on models, which permitted visualization and manipulation of the apices for necessary procedures. The models were placed in articulator to simulate the jaw. Six groups (G) were formed as: G1, G2 and G3 represented mandibular positioning of teeth and were irrigated with MNI, RE, and PUI, respectively, while G4, G5, and G6 represented maxillary positioning of teeth and were also irrigated in same sequence. Prior to the final irrigation, 72 cube-shaped foam pieces covered with aluminum foil were weighed and the values were recorded as the initial weights. The cubes were then placed on the apical part of each sample. Final irrigation was performed with distilled water and the cubes were weighed again to determine their final weight. Data were analyzed using Kruskal-Wallis and Mann-Whitney U post-hoc test (p<0.05).

**Results:**

Irrespective of the irrigation technique used, the amount of irrigant extruded from the apex showed a statistically significant difference related to the effect of gravity (p<0.05). There was no statistically significant difference between irrigation methods (p>0.05). When the irrigation systems were compared to examine the effect of gravity, the significant difference was found between G2 and G5 (p<0.05).

**Conclusions:**

Within the limitations of this study, MNI and PUI were found to be reliable irrigation systems. Caution should be exercised when using RinsEndo.

** Key words:**Final irrigation, manual needle irrigation, passive ultrasonic irrigation, RinsEndo.

## Introduction

Chemomechanical debridement is an important part of endodontic treatment. Many different irrigation techniques and devices have been used to improve disinfection of the root canal system. For optimal effectiveness of the irrigation, the irrigant should make direct contact with all parts of the canal wall (1). A flushing action, which is dependent on many factors such as the insertion depth, diameter of the needle (2), and the final size and taper of the prepared root canal (3), is necessary for optimal cleaning of the root canal (4). The conventional endodontic irrigation syringe and needle [manual needle irrigation (MNI)] is the most widely used technique because it is very easy to manipulate and affords good control of needle depth and the volume of irrigant delivered (5). However, it’s safety has been questioned because of the positive pressure used to introduce the irrigant into the canal, which could cause the solution to extrude into the periapex despite strict control of the working length (WL) and result in severe tissue damage and postoperative pain (6).

RinsEndo® (RE) (Dürr-Dental, Bietigheim-Bissingen, Germany) irrigates the canal by using pressure-suction technology. Employing a hydrodynamic working principle, its components include a handpiece, a cannula with a 7-mm-long exit aperture and a syringe carrying the irrigant. The handpiece is powered by a dental air compressor and has an irrigation speed of 6.2 mL/min.

It is well documented that ultrasound enhances the flushing action of irrigant solutions (7). The term ‘passive ultrasonic irrigation’ (PUI) was first defined by Weller *et al.* (8) “Passive activation” in this technique implies that the instrument, once inside the canal, does not touch the canal walls. During PUI, a small file or smooth wire (e.g., size 15) is placed at the center of the root canal after shaping the canal, and then activated ultrasonically to induce “acoustic streaming” (9). This increases the efficiency of cleaning by the irrigant inside the canal by means of hydrodynamic cutting power (10).

The purpose of this study was to compare the final amount of irrigant extruded apically due to MNI, RE, and PUI and assess the effect of gravity on irrigant extrusion using these techniques. There are limited studies examining the effect of gravity on apical extrusion of the irrigant (11,12). The null hypothesis was that the final amount of apically extruded irrigant would differ according to (i) irrigation technique and (ii) the effect of gravity.

## Material and Methods

-Sample Preparation

**Figure 1:**
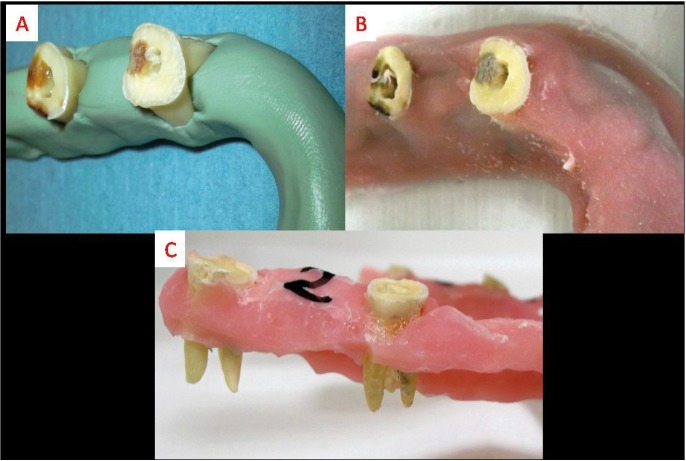
A) Specimens were placed in silicone impression material. B) The root/silicon set-up was surrounded with acrylic resin. C) Silicone material was removed and roots were exposed.

**Figure 2:**
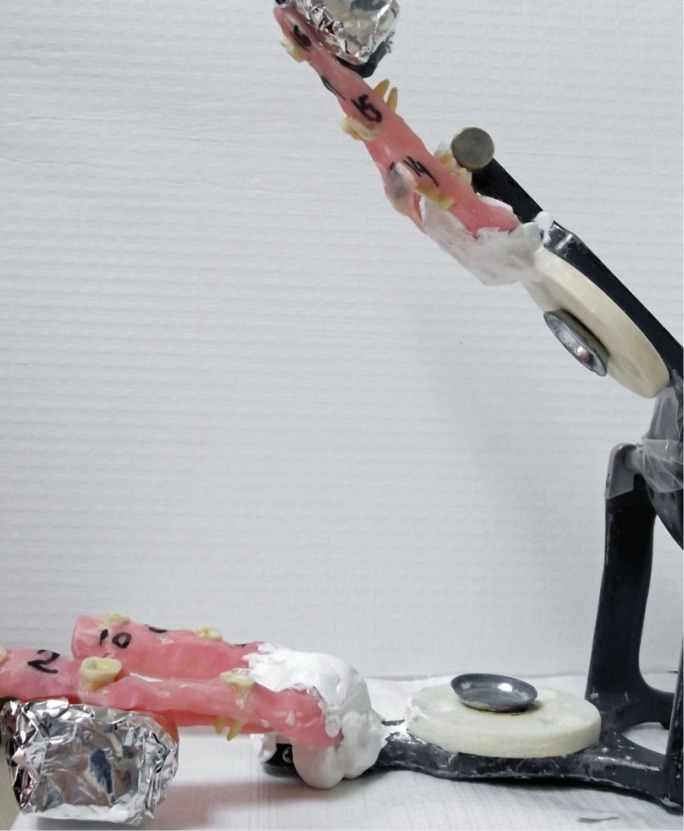
Proximal view of experimental set-up.

**Figure 3:**
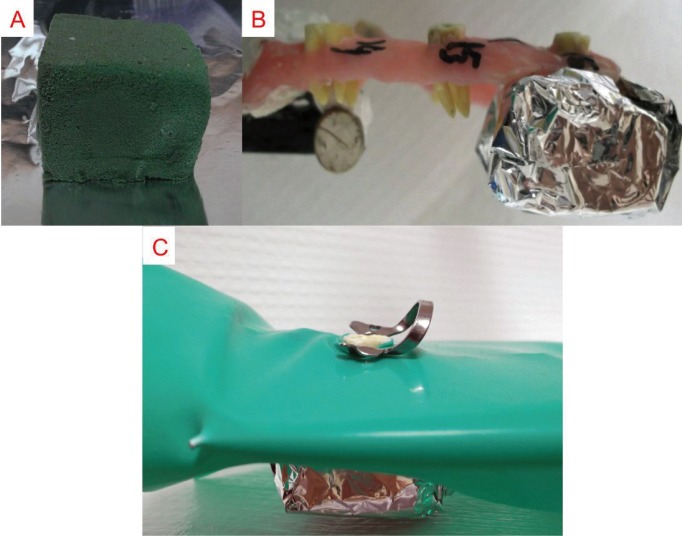
A) A piece of flower foam was placed inside foil. B) Foam covered with foil was placed on the apical part of the roots. C) Placement of rubber-dam during irrigation.

A total of 30 extracted maxillary molar teeth that had intact apices and had not been subjected to any previous endodontic treatment were collected and cleaned off to remove debris and soft tissue remnants. They then stored in 0.5% thymol solution until use. To reduce the effects of canal size and curvature on the extrusion of the irrigant, the distobuccal roots of all teeth were used as they had straight root canals of similar size. Teeth with a curvature between 0 and 10° were selected (13). To ensure similar lengths, all the teeth were measured and decoronated with a high-speed bur. An access cavity was then prepared in each tooth and the canal openings of the mesiobuccal and palatal roots were sealed with chemical composite resin (Alpha-Dent® Self Cure Composite, Dental Technologies, IL, USA). The WL was determined by introducing a size 10 K file (Mani, Inc., Utsunomiya Tochigi, Japan) into the canal until it was just visible at the foramen and then subtracting 1 mm from this measurement. The size of the minor constriction was controlled by introducing a size 15 K file (Mani, Inc., Utsunomiya Tochigi, Japan) up to the WL. Teeth in which the tip of the file extended beyond the apical foramen were excluded. As a result, the study was conducted with a final sample of 12 specimens. An operator then prepared all the distobuccal canals to the WL, up to size F2 (ISO size 25, taper 0.09-0.05) using ProTaper (Dentsply, Tulsa, OK) instruments. Between each file, the root canals were irrigated with 2 mL of 2.5% NaOCl solution using a syringe and a 27-gauge needle (Endo-Eze; 27-G, Endo-Eze, Ultradent South Jordan, UT). No specific attempts were made to remove the smear layer. Root canals were dried with paper points and the external surfaces of the specimens were carefully dried with air blasts. The study used a matrix design with three different irrigation methods and two positions. The same 12 teeth were used in all 6 groups so as to avoid variations in canal anatomy and apical diameter. First, the teeth were divided randomly into two groups (n=6) and embedded in horseshoe-shaped silicon impression material (Zhermack, BadiaPolesine (RO) – Italy) in a manner such that the roots were positioned inside the material while the crowns remained outside (Fig. 1A). The roots/silicon set-up was then surrounded with acrylic resin (Meliodent, HerausKulzer Ltd. Newbury, UK) up to the cementoenamel junctions of the teeth (Fig. 1B). After polymerization of the resin, the silicon impression material was removed with the help of a box cutter (Fig. 1C). The acrylic models, containing 6 teeth each, were then mounted on a semi-adjustable articulator with plaster so as to simulate the mandible and maxilla (Fig. 2). The mounting plate was parallel to the surface when irrigating the mandibular simulation. During irrigation of the maxillary simulation, the angle between the mounting plate and surface was maintained at nearly 45° in order to imitate the patient’s head position in the dental unit. In all groups, irrigation was performed with 10 mL of distilled water delivered to the pulp canal according to manufacturer’s instruction. Before irrigation, the operator placed a dental dam on the tooth to prevent observation of the extrusion (Fig. 3C). Immediately after each experiment, all specimens were dried with five size-25 paper points (Diadent; Chungcheongbukdo, Korea). The six experimental groups (G) were formed as follows: in G1, G2 and G3 acrylic model represented mandibular positioning of teeth and were irrigated with MNI, RE, and PUI, respectively, while in G4, G5, and G6 acrylic model represented maxillary positioning of teeth and were also irrigated in same sequence.

-Irrigation procedures

MNI: This technique was performed with a syringe and Endo-Eze Needle. The 27-G side-vented needle (Endo-Eze, Ultradent South Jordan, UT) was placed 2 mm short of the WL without any binding and moved in an up-and-down motion during irrigation. Hence, the root canals were irrigated for 1.5 min and the delivery rate was approximately 6.6 mL/min (14). Control of irrigation pressure was difficult in this group.

RE®: The irrigant was delivered at the rate of 6.2 mL/min and agitated by activation of the RE handpiece (Dürr-Dental, Bietigheim-Bissingen, Germany) using the needle provided by the manufacturer (needle size 45 with a lateral opening of 7 mm). The cannula was placed into the coronal third of the canal without any binding and moved up and down during irrigation. The compressed air pressure supplying the handpiece was adjusted to 3.5 bar to ensure it was within the recommended range (2.3–4.2 bar). The root canals were irrigated for 1.6 min (14).

PUI: PUI was performed with a stainless steel #15/.00 file (IrriSafe K15; Satelec, Merignac, France) driven by an ultrasonic device (SuprassonPMax; SatelecActeon, Merignac, France) with in-plane oscillation in the direction of the root canals. An IrriSafe ultrasonically activated file on a Satelec P5 booster ultrasonic unit (Satelec) with power setting 5 was placed 1 mm short of the WL and activated. During PUI, the root canals were irrigated for 1 min with a continuous flush of the irrigant (10 mL/min) (14). Every attempt was made to keep the file centered in the canal so as to minimize contact with the canal walls, as any such contact could dampen the oscillatory motion of the file.

A cube-shaped piece of floral foam covered in foil was attached to the root tip of every tooth, so as to simulate the slight resistance of periapical tissues and prevent the loss of extruded water (Fig. 3 A,B) (15). For each measurement (n=72), one piece of floral foam covered with foil was weighed prior to any procedures with the help of a 0.0001 electronic balance (Sartorius basic, Sartorius AG, Gottingen, Germany). Three consecutive measurements were taken and their mean was considered to be the weight of each foam piece. If these 3 readings showed great variability, the process of weighing was continued until 3 similar measurements were obtained (where only the last digits differed by 1–2). The electronic balance was placed in a room without any windows and the door of the room was closed during weighing of the cubes. Only two individuals, who were seated and motionless during the process, were present in the room. To monitor the machine’s precision, the same 3 foam pieces were weighed at every stage. During irrigation, the root tips were pressed into the foam. The foam pieces were weighed again after irrigation was completed and the mean of these measurements was considered to be the new weight. The amount of apically extruded irrigant was calculated by subtracting the initial recordings from the final weight of the foil-covered foam cubes. As the experiment was conducted at room temperature with water as the irrigant, no conversion between the weight and volume was performed because the specific gravity of water at 25°C (77°F) is 1.00, up to the second decimal place (16). The differences between groups were analyzed using Kruskal-Wallis and Mann-Whitney U post-hoc test (*p*<0.05).

## Results

The mean weights and standard deviations for each group are presented in  Table 1. The results indicated that all of the irrigation techniques caused measurable apical extrusion of irrigant. Irrespective of the irrigation methods, the final amount of irrigant extruded showed a statistically significant difference related to the effect of gravity (*p*=0.002). Irrespective of tooth position, there was no significant difference between irrigation methods (*p*=0.090). In contrast to PUI and MNI, RE showed much greater extrusion in both positions. In comparisons within groups, only RE showed a significant difference in the amount of irrigant extruded related with the effect of gravity (*p*=0.045). In the mandibular and maxillary positions, all the techniques showed the same extent of apical extrusion of irrigant (*p*>0.05).

**Table 1:**
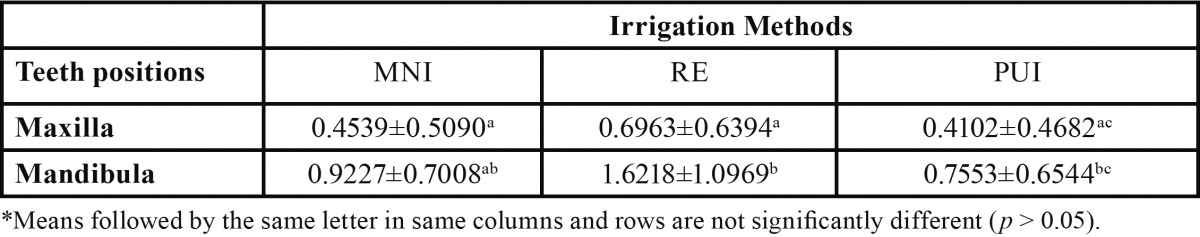
Mean and standard deviation of extruded irrigant for each group (gram)*.

## Discussion

During root canal instrumentation, pulp tissue remnants, dentine debris, microorganisms, and intracanal irrigants may be extruded from the apical foramen and induce flare-ups. It is known that inflammatory reactions can cause bone resorption, edema and postoperative pain (6,17). While it is important to ensure that the irrigant penetrates the entire root canal system in order to enable it to exert its favorable actions (18), it is also necessary to ensure that it does not extrude into the periapical tissues. This helps maintain the critical balance between cleaning efficacy and patient safety (19).

The results of this study can only be generalized to teeth with fully formed apices and straight root canals. Repeated use of the same specimens had a negligible effect on the results under the experimental conditions used (20). Psimma *et al.* (20) reported that an increase in the constriction diameter is linked to a slight increase in irrigant extrusion, but the difference was not statistically significant. In the present study, a standardized constriction was created in all the specimens so as to avoid the confounding effect of this factor.

In experimental studies, the amount of material extruded from the apex is usually between 0.1 g and 1.2 g. VandeVisse and Brilliant (21) reported that a collectible amount of debris was extruded only when root canal instrumentation was accompanied with irrigation. In this study, NaOCl was used for irrigation during preparation and after drying the canals with paper points; final irrigation was performed with distilled water (16). The root tips were pressed into floral foam only at this stage, and the extruded irrigant values were recorded, which have been presented in  Table 1.

Most of the literature regarding extrusion studies involves teeth that had their apices positioned downwards in the vial, representing a mandibular tooth. However, gravity may have an effect on the amount of solution extruded from the apex as it may influence the accessibility of the irrigation solutions to the apex. Currently, only two studies have investigated the effects of gravity on extrusion from the root apex (11,12). In present study, irrigant extrusion was observed even in the maxillary position despite the fact that it was against gravitational force, similar to the results found in previous studies (11,12). There was a significant difference between extrusions in the two positions irrespective of the irrigation technique used. In the current study, significantly larger quantity of irrigant was extruded in the mandibular position compared to the maxillary position. The results of this study were consistent with those obtained by Williams *et al.* (11). On the other hand, the effect of gravity on the apical extrusion of irrigant may not be important as the patient is usually in a supine position, except in some special situations such as pregnancy and spinal deformities (kyphosis).

It has been recommended that the needle tip be placed 2 mm short of the WL or slightly coronal to the point when resistance is encountered, before the needle tip reaches the desirable distance (22). Therefore, in the present study, the same needle penetration depth was used. A previous study performed using a Computational Fluid Dynamics model showed that a flat needle (open-ended needle) led to a higher mean pressure at the apical foramen than the double side-port needles, at the same depth (23). Thus, side-vented needles were used in this study.

The results of this study agreed broadly with those of previous studies in which irrigation with positive pressure was observed to result in periapical extrusion (16,24). Several studies examined the efficacy of RE in comparison with manual and ultrasonic irrigation (14,16,25). Desai and Himel (16) reported periapical extrusion of the irrigant for MNI, RE and also PUI. The manufacturer’s instructions for RE suggest that the hydrodynamic activation of the irrigant ensures that the apical third of the root canal is effectively irrigated, even though the needle tip is inserted into the coronal third. The results of present study confirmed this. Similar to previous study, it was demonstrated that RE extruded significantly more irrigant from the root canal despite the fact that the needle tip was placed the most coronally (16). Hauser *et al.* (25) reported that the penetration depth of a colored irrigant into the root canal dentine was higher for RE when compared with syringe irrigation, demonstrating the efficacy of the oscillation in distribution of the irrigant. However, PUI extruded significantly less irrigant than RE in the mandibular position, although the file was 1 mm shorter than the WL. Maximum attention was paid to avoid any contact of the instrument with the canal walls during the PUI procedure. According to the results of a previous study, the amount of irrigant extruded apically during PUI is fairly little (26). Tasdemir *et al.* (26) reported that the use of a passively activated file with ultrasonics in a canal after instrumentation resulted in a low risk of apical extrusion of the irrigating solution. In the present study, PUI extruded less irrigant than the MNI, although the difference was not significant. In the MNI group, the control of irrigation pressure was difficult and this could have affected the amount of irrigant extruded into the periapical tissues.

Martin *et al.* (27) demonstrated the ability of ultrasonically activated K files to cut dentin. The roots used in the present study were straight and PUI was performed after the root canals had been shaped. As stated previously, every attempt was made to keep the file centered in the canal so as to minimize contact with the canal walls. Moreover, PUI was performed last in all the groups to prevent any alteration of the root canal shape, similar to the procedure used by Rodig *et al.* (14).

Most of the previous studies used a vial attached to the apices of the teeth to collect the extruded debris and irrigant (16,26) and measured the mean extruded debris after evaporation of the irrigating solution. However, in this study, the total amount of extruded material during final irrigation was measured. No special attempt was made to distinguish the amount of debris from the amount of irrigant as they are both responsible for periapical inflammation, postoperative pain, and possible delayed healing (24). Altundasar *et al.* (15) reported that the resistance provided by the floral foam may be more realistic than the assumption of zero back-pressure, which is common in extrusion studies that use a vial setup with no periapical resistance. No significant differences were found in the weights of the floral foams during the study, which demonstrated that precise measurement was possible with the balance used in the study. However, the results may vary in an in vivo model because of the presence of periapical tissues, which act as a natural barrier to prevent irrigant and debris extrusion.

In conclusion, the null hypothesis of the study was accepted. The degree of apical extrusion of irrigant was dependent on the type of irrigation technique and gravity. Greater caution should be taken during irrigation so as to prevent postoperative pain. Among the three techniques used, RE was responsible for the heaviest amount of extruded debris, especially in the mandible.
